# Progress in Probiotic Science: Prospects of Functional Probiotic-Based Foods and Beverages

**DOI:** 10.1155/ijfo/5567567

**Published:** 2025-04-14

**Authors:** Musaalbakri Abdul Manan

**Affiliations:** Food Science and Technology Research Centre, Malaysian Agricultural Research and Development Institute (MARDI), MARDI Headquarters, Persiaran MARDI-UPM, Serdang, Selangor, Malaysia

**Keywords:** *“future foods*”, “*do more good than harm*”, “*friendly bacteria*”, “*super probiotics*”, lactic acid bacteria (LABs), probiotic-based foods and beverages

## Abstract

This comprehensive review explores the evolving role of probiotic-based foods and beverages, highlighting their potential as functional and “*future foods*” that could significantly enhance nutrition, health, and overall well-being. These products are gaining prominence for their benefits in gut health, immune support, and holistic wellness. However, their future success depends on addressing critical safety concerns and navigating administrative complexities. Ensuring that these products “*do more good than harm*” involves rigorous evaluations of probiotic strains, particularly those sourced from the human gastrointestinal tract. Lactic acid bacteria (LABs) serve as versatile and effective functional starter cultures for the development of probiotic foods and beverages. The review emphasizes the role of LABs as functional starter cultures and the development of precision probiotics in advancing these products. Establishing standardized guidelines and transparent practices is essential, requiring collaboration among regulatory bodies, industry stakeholders, and the scientific community. The review underscores the importance of innovation in developing “*friendly bacteria*,” “*super probiotics*,” precision fermentation, and effective safety assessments. The prospects of functional probiotic-based foods and beverages rely on refining these elements and adapting to emerging scientific advancements. Ultimately, empowering consumers with accurate information, fostering innovation, and maintaining stringent safety standards will shape the future of these products as trusted and beneficial components of a health-conscious society. Probiotic-based foods and beverages, often infused with LABs, a “*friendly bacteria*,” are emerging as “*super probiotics*” and “*future foods*” designed to “*do more good than harm*” for overall health.

## 1. Introduction

Functional probiotic-based foods and beverages fall into distinct categories based on their intended functions and advantages, directly linked to the specific probiotic microbes they contain. This category includes products that extend beyond basic nutrition, offering unique health benefits attributed to their specialized ingredients. Probiotic components find their way into diverse food and beverage applications, spanning dairy products, nondairy beverages, cereals, and even infant formula. The flourishing awareness among consumers about the potential of probiotics is steadily gaining momentum. This rise in awareness is poised to fuel increased investments in research and development endeavours aimed at creating novel products infused with probiotic elements. This anticipated drive-in research and development signifies an industry-wide focus on innovating and diversifying probiotic-based offerings to cater to evolve consumer demands and preferences.

In recent years, a profound shift has occurred in global dietary preferences, a shift characterized by an ever-increasing demand for functional foods [1]. This movement has been encouraged by growing health concerns worldwide, as populations grapple with escalating rates of chronic diseases and a heightened awareness of the important role nutrition plays in overall well-being [2]. As a result, the horizon for functional foods, particularly probiotic-based options, has expanded significantly. Among this landscape of health-conscious choices, the profound impact of the COVID-19 pandemic has propelled the interest in functional probiotic-based foods and beverages to the forefront of nutritional discourse [3, 4]. The pandemic, with its far-reaching consequences and the absence of a definitive cure or treatment for the SARS-CoV-2 virus, has prompted a re-evaluation of dietary interventions [5–8]. According to Koirala and Anal [8] and Shi et al. [9], probiotic-based foods and beverages stand out among the emerging functional foods in the market, marked as “*future foods*” due to their increasing popularity and broad acceptance among consumers. The potential of these “*future foods*” as effective nutritional therapy and biotherapeutics has garnered widespread attention.

As per the latest findings from Research and Market in August 2023, the global probiotics market is on a trajectory poised for substantial growth. Projections indicate an impressive surge, with the market expected to expand to a staggering $220.14 billion by the year 2030. This exponential growth signifies a notable compound annual growth rate (CAGR) of 14.0% from 2023 to 2030. Numerous factors contribute to this steady ascent within the market landscape. Shifting dietary preferences toward healthier food alternatives are significantly fuelling this growth. Consumers worldwide are increasingly gravitating toward foods and beverages known for their health-enhancing properties, particularly those promoting digestive health. This fundamental shift in consumer behaviour, coupled with a rising awareness of the benefits associated with digestive health products, is driving the continuous increase in the probiotics market. The growing awareness among consumers about the important role of digestive health in overall well-being has sparked a heightened interest in probiotic-rich products. This growing consumer consciousness, paired with the preference toward more health-conscious dietary choices, serves as a robust catalyst propelling the ongoing expansion of the global probiotics market.

In addition, a recent Research and Market Survey released in December 2023 found that the global probiotics market was valued at approximately $59.3 billion in 2022. Projections indicate a substantial growth trajectory, with estimates foreseeing a significant expansion to approximately $91.7 billion by 2030. This growth trend is expected to manifest at a CAGR of 5.6% during the analysis period spanning from 2022 to 2030. Among the segments analysed in the report, bacteria emerge as an important sector poised for notable advancement. Projections suggest a steady growth rate, with a projected CAGR of 5.3%, culminating in an estimated value of $70.8 billion by the culmination of the analysis period. The yeast segment, undergoing readjustments considering ongoing postpandemic recovery, is anticipated to experience robust growth in the coming 8 years. The revised projections indicate a revised CAGR of 6.8% for this segment, reflecting a promising increase in the global market. These forecasts consider the recovery phase post the pandemic, indicating a renewed vigour in the market dynamics. The anticipated growth rates underscore the resilience of the probiotics market, signalling significant opportunities for expansion and innovation within the industry.

At a time when the world faces the uncertainties of a viral adversary, the concept of leveraging dietary choices to fortify one's immune system and overall health has taken on paramount importance [10, 11]. Probiotics, long celebrated for their potential benefits to gut health and immunity, have emerged as a beacon of hope [12, 13]. Their ability to potentially bolster the body's defences and support the gut microbiome has ignited a rise in research and innovation within the realm of probiotics for food products. The absence of a definitive cure for SARS-CoV-2 has underscored the urgency for alternative measures to enhance immune resilience [5, 7]. Functional probiotic-based foods and beverages, recognized for their potential to modulate the gut microbiota and potentially boost immunity, have stepped into the limelight as a promising avenue for nutritional intervention [5, 14]. With the conventional pharmaceutical solutions yet to yield a conclusive cure, the exploration of dietary options like probiotics has gained momentum.

The surge in interest is not solely due to the traditional applications of probiotics but also the exploration of next-generation strains and their potential benefits. The prospect of these novel strains contributing to improved health outcomes has further fuelled research and innovation, marking a transformative phase in the landscape of functional probiotic-based foods and beverages. The horizon seems ripe for the emergence of a variety of food applications integrating these beneficial microorganisms. Among this growing landscape of probiotics as “*future foods*,” safety assumes paramount importance. While the fundamental premise guiding the application of probiotics in foods has historically been to “*do more good than harm*,” the proliferation of novel strains and associated safety issues necessitates a precise evaluation process. Strains sourced from the human gastrointestinal tract, absence of pathogenicity and virulence factors, nonpromotion of disease-related activities, and the avoidance of transferrable antibiotic resistance genes form the cornerstone of safety considerations [15, 16].

However, safety considerations alone are not the solitary challenge in this domain. The administrative framework governing functional probiotic-based foods and beverages requires equal attention. Across various countries and regions, divergent sets of legislations, policies, and governmental guidance documents prevail. Harmonizing these frameworks while safeguarding consumers from misleading claims poses a formidable challenge. Clinical evidence and approvals remain challenging to obtain, leading to varying standards and regulations across jurisdictions [17–19]. Despite continuous improvements in standards, policies, and regulations, the rapidly growing market of functional probiotic-based foods and beverages demands more demanding efforts to assure consumer safety [20]. The confluence of health considerations, scientific advancements, market dynamics, and administrative intricacies signifies a complex landscape that necessitates a delicate balance between innovation and consumer well-being.

This comprehensive review navigates the intricate landscape of probiotics, exploring their historical applications, the emergence of novel strains, and their important role in enhancing health and well-being. It sheds light on crucial safety considerations, underscoring the significance of sourcing strains from the human gastrointestinal tract, mitigating pathogenicity and virulence factors, and averting antibiotic resistance genes. The review further delves into the challenges associated with obtaining clinical evidence and administrative approvals for probiotics, resulting in varying standards and regulations across different regions. Emphasizing the dynamic nature of the probiotics market, it stresses the continuous need for improvement in standards, policies, and regulations to keep pace with its rapid growth. A key focus of the review is on the complexities inherent in gaining approvals and the necessity for global harmonization to ensure consumer safety and encourage innovation. Through advocating collaborative efforts among administrative bodies, industry stakeholders, and the scientific community, the review envisions a future where probiotic-based foods and beverages stand as trustworthy, effective, and integral components of a health-conscious society.

## 2. The Evolution of Probiotics in Foods

The historical tapestry of probiotics in foods unveils a rich legacy woven into diverse cultures across centuries. From ancient fermentation practices to modern innovations, the use of probiotics spans civilizations, each with its unique repertoire of traditional strains. Centuries ago, our ancestors intuitively recognized the transformative power of fermented foods. These age-old practices involved the intentional fermentation of various foods using natural bacteria, giving rise to iconic probiotic-rich staples. The historical usage of probiotics in food traces back centuries, rooted in various cultures' culinary practices.

Yogurt, among the earliest probiotic foods, originates from ancient Mesopotamian and Indian cultures. Its health-promoting properties were documented as far back as 6000 bc in Indian Ayurvedic scripts [21]. Legend has it that yogurt likely originated from the fermentation process that occurred in animal skin bags used for transporting water and milk in the hot, dry climates of Middle Asia and the Middle East [22]. It was not until the 20th century that Stamen Grigorov (1878–1945), a Bulgarian medical student, discovered lactic acid bacteria (LABs) as the key to these benefits [21]. Elie Metchnikoff is recognized for pioneering the idea that live microorganisms can promote health by manipulating the gut microbiome with beneficial bacteria from yogurt, introducing the concept of probiotics in medicine. He also suggested introducing LABs, including Lactobacilli from Bulgarian yogurt cultures [23–25]. Isaac Carasso (1874–1939) recommended yogurt to his patients suffering from gastrointestinal issues. Following this, he began producing yogurt and founded the Danone Company in 1919 [22, 26]. Numerous scientific articles, historical texts, and archaeological findings discuss the use of yogurt and fermented milk as early probiotics due to their microbial content and purported health advantages ([27, 28].  Table 1 provides a summary of the history of probiotic concepts or definitions.

Similarly, fermented vegetables, prevalent in Asian cultures like Korea and Japan, introduced probiotic strains through the process of lactofermentation. Sauerkraut, kimchi, yogurt, kefir, kombucha, tempe, natto, and miso emerged as probiotic powerhouses, marrying preservation with health-enhancing properties. These traditional strains, passed down through generations, were cherished for their digestive benefits and believed to contribute to overall health. However, the landscape of probiotics in food has evolved significantly. Beyond these traditional strains, the exploration of emerging probiotic strains has gained prominence. The hypothesis suggests that higher levels of bifidobacteria in the infant gut could be linked to the health benefits observed in breast-fed infants compared to those fed with formula [45]. Scientific advancements and technological innovations have facilitated the identification and isolation of novel strains with potential health-enhancing properties.

## 3. Exploration of Functional Probiotics in Foods and Beverages

The exploration of probiotics in foods and beverages unveils a world of microscopic superheroes. These beneficial microorganisms, found in fermented foods and emerging novel strains, play an important role in promoting health and well-being. From historical practices to cutting-edge research, this journey delves into the significance of probiotics sourced from the human gut, their potential to support immune function, and their intricate relationship with gut health [46–49]. Understanding the role and impact of these “*friendly bacteria*” in our diet paves the way for a holistic approach to nutrition and wellness.

While LABs have traditionally dominated probiotic food and beverage formulations, growing research highlights the significance of *S. cerevisiae* var. *boulardii* as a probiotic yeast with promising functional applications [50]. This yeast strain has demonstrated remarkable resistance to the acidic environment of the gastrointestinal tract and plays a crucial role in enhancing intestinal microbiota balance, making it an important addition to probiotic formulations [34]. Recent studies suggest that *S. cerevisiae* var. *boulardii* can be successfully incorporated into various food and beverage products, expanding the scope of functional probiotic applications [51]. Yeast species such as *Saccharomyces*, *Candida*, *Debaryomyces*, *Yarrowia*, and *Kluyveromyces* have been identified for their probiotic potential and are commonly introduced into dairy products like kefir, yogurt, kumis, and cheese, where they enhance probiotic functionality and sensory attributes in fermented dairy products such as yogurts and fermented milks [52, 53]. Additionally, its application in fermented alcoholic beverages, including beers, wines, and meads, has gained interest due to its ability to enhance fermentation efficiency and flavor complexity. Beyond dairy and alcoholic products, probiotic yeast is also being explored in nonalcoholic fermented beverages, vegetable-based products, and baked goods, creating new possibilities for probiotic-enriched foods [34]. The integration of *S. cerevisiae* var. *boulardii* in food development offers a holistic approach to probiotic innovation, complementing the benefits of LABs while providing unique functional, sensory, and fermentation advantages. As consumer demand for diverse probiotic sources grows, the inclusion of probiotic yeasts in functional food and beverage development is an emerging trend that holds great potential for future research and commercialization. Among the types of products listed under functional probiotic-based foods and beverages widely discussed in the open literature are those presented in Table 2.

## 4. Probiotics and Their Role in Promoting Health and Well-Being

Probiotics are live microorganisms, often referred to as “*friendly bacteria*,” that confer a myriad of health benefits when incorporated into our diet [104, 105]. Genera like *Bifidobacterium* or *Lactobacilli*, known for producing lactic acid, have long been linked to good health [106], Additionally, *Saccharomycetes*, *Bacillus*, and other strains that can meet the basic conditions of probiotics become probiotics [104, 107, 108]. Increasing these bacteria in the body can be achieved by administering suitable strains as probiotics or by supplying prebiotic growth substrates to individuals [109–111]. Their important role lies in cultivating a harmonious gut environment, where they bolster the balance of our intestinal microbiota. This equilibrium in our gut flora is important for efficient digestion, nutrient absorption, and immune system modulation. Probiotics act as the guardians of our gastrointestinal health, aiding in the breakdown of complex carbohydrates, producing essential vitamins, and fortifying our gut against harmful pathogens [112, 113]. Moreover, their influence extends beyond the gut, impacting various aspects of our health. Studies suggest their role in potentially alleviating symptoms of certain gastrointestinal disorders, reducing the risk of infections, and even contributing to mental well-being [16, 114]. The intricate relationship between the gut and the brain, known as the gut–brain axis, hints at probiotics' potential to influence mood and cognitive function [115, 116].

Their ability to modulate the immune system adds another layer to their significance. Probiotics engage in a delicate dance with our immune cells, fine-tuning their responses and enhancing our body's defence mechanisms. Probiotics function through three main mechanisms: (1) boosting the host's defence capacity, (2) directly combating microorganisms, and crucially (3) the function of metabolites [104, 117]. This interaction plays a crucial role in reducing the risk of certain allergies, infections, and inflammatory conditions. In essence, probiotics emerge as unsung heroes within our bodies, diligently working to promote a state of balance and vitality. Their role in supporting digestive health, fortifying our immune system, and potentially impacting mental wellness underscores their profound significance in promoting overall health and well-being [10, 11, 111]. Comprehensive studies affirm that the vitality of probiotic colonies significantly impacts the functionality of virtually every physiological system within the human body [28].  Table 3 succinctly delineates the beneficial effects of probiotics on humans, derived from an open literature review.

### 4.1. The Benefits of Probiotics From the Human Gut

Scientific investigations have elucidated the important contribution of gut microbiota to the holistic well-being of various anatomical components, exerting profound effects on host physiology. Notably, their impact extends to the immune system, wherein these microorganisms intricately participate in the development and functionality of immune cells [7, 290]. Their modulatory influence manifests through the augmentation of tolerance levels, reinforcement of pathogen resistance, and mitigation of inflammatory responses [291, 292]. Within the intricate framework of the nervous system, symbiotic microorganisms exhibit an indirect facilitation of neurodevelopment and a concomitant reduction in behavioural disorders while concurrently promoting serotonin production [293–295]. Empirical investigations underscore the substantial therapeutic potential of probiotics. The discernment of the precise mechanisms of action employed by distinct beneficial bacterial strains holds promise for their utilization as natural and safe alternatives or adjuncts to pharmacological interventions, with the aim of preventing and improving symptoms across various conditions. The integration of probiotics into functional foods and beverages further enhances their feasibility for seamless incorporation into daily dietary practices [1, 296].

Probiotics sourced from the human gastrointestinal tract emerge as nature's blueprint for gut health and well-being [112, 113]. These beneficial bacteria, indigenous to our own bodies, wield profound significance in nurturing a balanced gut microbiome and influencing overall health. The human gastrointestinal tract, a bustling ecosystem teeming with diverse microbial communities, houses a plethora of probiotic strains uniquely suited to our physiology [16, 113]. These endogenous probiotics play an important role in maintaining gut homeostasis, bolstering the intestinal barrier, and modulating immune responses. Their significance lies not just in their presence but in their functionality. These indigenous probiotics engage in a delicate dance within the gut, aiding in the breakdown of food, fermenting fibres, and producing bioactive molecules and essential compounds against pathogens such as antimicrobial metabolites, including bacteriocins, vitamins, SCFAs, enzymes, amino acids, organic acids, peptides, sugar polymers, hydrogen peroxide (H_2_O_2_), and biosurfactants [104, 297, 298]. Moreover, they outcompete harmful pathogens, acting as a frontline defence mechanism against gut infections. Probiotics have undergone scrutiny for their effectiveness in managing a wide array of gastrointestinal disorders, enhanced immunity, and improved metabolic health [112, 130, 177]. These encompass antibiotic-associated diarrhea (including *Clostridium difficile*–associated intestinal disease) and various infectious bacterial and viral diarrhea types caused by rotavirus, *Shigella*, *Salmonella*, enterotoxigenic *Escherichia coli*, *Vibrio cholerae*, and human immunodeficiency virus/acquired immunodeficiency disorder, among others [112]. Additionally, they have been explored in conditions like enteral feeding diarrhea, *Helicobacter pylori* gastroenteritis, sucrase maltase deficiency, inflammatory bowel disease, irritable bowel syndrome, small bowel bacterial overgrowth, and lactose intolerance [112, 170, 177, 299, 300]. Probiotics have the ability to block the action of intestinal bacterial enzymes responsible for creating colonic carcinogens [112, 301]. Probiotics contribute to intestinal health through various means, such as boosting immunity, competing for nutrients, preventing adherence to the epithelial and mucosal surfaces, hindering invasion of the epithelium, and generating antimicrobial substances [112, 235, 276, 290].

Rolfe [112] highlights that the advantages of probiotics originating from the human gastrointestinal tract go well beyond gut-related effects. Their interplay among enteric microbiota, the central and enteric nervous systems within the gut–brain axis suggests a capacity to impact cognitive function and mental health [302]. According to [302], the bidirectional interaction between microbiota and the gut–brain axis involves signalling from the gut microbiota to the brain and vice versa, facilitated through neural, endocrine, immune, and humoral connections. Gao et al. [207] showcase the broader impact of probiotics and intestinal microbiota homeostasis. They contribute to regulating systemic inflammation, influencing conditions not limited to the digestive system. These encompass allergies, skin health (including aging-related concerns like wrinkles, pigmentation, and dryness), and metabolic disorders, revealing the existence of a gut–skin axis. Harnessing the potential benefits of these endogenous probiotics entails nurturing a favourable environment within the gut [112]. Factors like environment, diet, age, lifestyle, and exposure to antibiotics can impact the balance of these beneficial microbes [104, 303]. Thus, fostering an environment conducive to their proliferation becomes important for reaping their rewards. In essence, probiotics sourced from our gastrointestinal tract are not just inhabitants; they are integral players orchestrating a symphony of health within us [113]. Their role in maintaining gut health, fortifying immunity, and potentially influencing systemic well-being underscores their profound significance in optimizing our overall health.

### 4.2. How Probiotics Support Immune System and Gut Health

Probiotics, those microscopic superheroes inhabiting our gut, wield a dual power in fortifying our immune system and maintaining gut health. Their important role in these domains underscores their significance as key players in our overall well-being. Primarily, probiotics act as guardians of our gut, fostering a harmonious balance within our intestinal microbiota. This balance is crucial for gut health, as it influences digestion, nutrient absorption, and the integrity of the gut lining. By outcompeting harmful bacteria and modulating the gut's environment, probiotics help maintain a robust gut barrier, preventing the infiltration of pathogens and reducing the risk of gastrointestinal infections [113, 235]. Moreover, their influence extends beyond the gut walls, influencing our body's immune response. Probiotics engage in a dynamic interplay with our immune cells, modulating their activity and enhancing their functionality [290]. This interaction helps regulate the immune system's responses, ensuring it reacts appropriately to threats without overreacting, thereby reducing the risk of allergies, inflammatory disorders, and infections [49, 236, 237].

The gut–immune axis, a complex network connecting the gut and immune system, highlights the intimate relationship between probiotics and immunity [304]. The communication between these two systems is important for overall health. Probiotics' ability to fine-tune this communication is key to maintaining a balanced immune response and promoting overall immune health. Harnessing the potential of probiotics to bolster the immune system and support gut health involves fostering an environment conducive to their proliferation [238–240]. Factors such as dietary choices rich in prebiotics, lifestyle modifications, and minimizing the use of antibiotics can positively impact the balance of these beneficial microbes, enhancing their effectiveness in promoting gut health and fortifying immunity [305]. In essence, probiotics emerge as versatile allies, playing a crucial role in maintaining a robust gut environment and modulating immune responses. Their intricate relationship with both gut health and immune function underscores their significance as integral components of our overall health and well-being [178–180].

The benefits of probiotics sourced from the human gastrointestinal tract are multifaceted. These probiotic strains have the potential to restore and maintain a balanced gut microbiota, essential for proper digestion and nutrient absorption [5, 306]. They aid in the breakdown of complex carbohydrates, production of essential vitamins like B and K, and synthesis of certain enzymes crucial for gut health [48]. Moreover, the symbiotic relationship between probiotics and the immune system is a subject of extensive research. Probiotics are believed to modulate immune responses by influencing the gut-associated lymphoid tissue (GALT) [290, 307]. This interaction plays an important role in bolstering the immune system, potentially reducing the risk of infections and allergic reactions.

The intricate balance of the gut microbiota is closely linked to immune function [5, 14]. Probiotics' ability to support this balance may contribute to fortifying the body's defences against pathogens [290]. Studies suggest that certain probiotic strains stimulate the production of immune-regulating substances, enhancing the body's ability to mount an appropriate response to external threats [276]. Furthermore, probiotics are believed to have anti-inflammatory properties, playing a role in mitigating chronic inflammation, an underlying factor in various health conditions. By maintaining a healthy gut environment, probiotics may help alleviate symptoms of certain IBDs like Crohn's disease and ulcerative colitis [112, 114]. Additionally, probiotics' potential in promoting mental health is an area of burgeoning interest [308]. The gut–brain axis, a bidirectional communication network between the gut and the brain, has led to investigations into how probiotics might positively influence mental well-being [293, 294, 309]. Preliminary studies suggest a correlation between gut health and mental health, hinting at the role of probiotics in managing conditions like anxiety, depression, and other mental disorders as well as the protective effects of dietary components [122, 310]. The exploration of probiotics in food unveils a diverse array of strains, both traditional and emerging, with the potential to profoundly impact health and well-being [1]. Sourcing strains from the human gastrointestinal tract amplifies their efficacy in supporting gut health, modulating immune responses, and potentially influencing mental health [114, 306]. As research continues to unravel the multifaceted benefits of probiotics, their integration into dietary practices represents a promising avenue for fostering holistic health.

### 4.3. Increasing Popularity of Functional Probiotic-Based Food and Beverage Dietary Supplements

The increasing popularity of functional probiotic-based foods and beverages stems from a convergence of drivers reshaping dietary preferences and health-conscious choices worldwide. These drivers underscore a rapidly growing interest in these products, fuelled by evolving consumer attitudes and a growing emphasis on holistic wellness. Consumer awareness and education play an important role in propelling the popularity of functional probiotic-based foods and beverages. Heightened awareness of gut health's significance and the role of probiotics in fostering a balanced microbiome has prompted consumers to seek out these functional foods [5, 6, 311]. Media coverage, educational campaigns, and information dissemination have empowered individuals to make informed choices, driving the demand for these products. A shift toward preventive health measures and a proactive approach to wellness has spurred the uptake of dietary supplements, including functional probiotic-based foods and beverages [312, 313]. The desire to fortify the immune system, improve digestive health, and promote overall well-being aligns with consumers' aspirations for a healthier lifestyle [238–240].

Moreover, the diversification and innovation in the food and beverage industry have contributed to the proliferation of probiotic-based products. From yogurts to kombucha, these functional foods now span a wide spectrum of options, catering to varying tastes and dietary preferences. This variety appeals to a broader consumer base, further driving the market's growth. The influence of health practitioners and scientific endorsements cannot be overlooked. Recommendations from healthcare professionals, along with scientific studies highlighting the benefits of probiotics, lend credibility and foster consumer trust in these products [314]. As a result, consumers increasingly integrate functional probiotic-based foods and beverages into their daily dietary routines. The increasing popularity of functional probiotic-based foods and beverages is underpinned by a confluence of factors, including heightened consumer awareness, a proactive approach to wellness, product diversification, and professional endorsements. This surge in demand reflects a growing recognition of the potential health benefits these products offer, indicating a continued trajectory toward a more health-conscious society.

## 5. Safety Considerations in Functional Probiotic-Based Foods and Beverages

The fundamental principle guiding the incorporation of probiotics into foods encapsulates a simple yet profound notion: “*do more good than harm*.” This principle underscores the essence of leveraging these beneficial microorganisms to enhance health outcomes while ensuring that their inclusion in food products does not pose any adverse effects on consumers. However, as the landscape of functional probiotic-based foods and beverages evolves, safety considerations assume critical importance, especially considering the emergence of novel strains and their potential implications [315]. The advent of novel probiotic strains has sparked heightened awareness regarding safety concerns. The evaluation of these emerging strains necessitates stringent criteria to ascertain their safety and efficacy. Safety concerns predominantly revolve around the potential risks associated with the introduction of new microbial strains into food products, particularly considering their intended consumption by the general populace [316].

### 5.1. Safety Evaluation

The safety evaluation criteria for probiotics encompass several key aspects. First and foremost is the origin of the strains. Strains sourced from the human gastrointestinal tract are deemed more compatible with the human body, exhibiting higher survival rates in the intestinal environment [114]. This criterion is important in ensuring that the introduced strains align with the natural microbiota of the gut, enhancing their effectiveness without causing disruptions [14]. Furthermore, the absence of pathogenicity and virulence factors in probiotic strains is imperative. Pathogenicity refers to the ability of microorganisms to cause disease, while virulence factors are attributes that enhance a microbe's ability to cause harm [317]. Ensuring that probiotics lack these detrimental traits is essential to prevent any potential adverse effects on consumers' health.

Another critical safety consideration is the avoidance of transferrable antibiotic resistance genes. The rise of antibiotic resistance is a global health concern, and the transfer of resistance genes from probiotics to pathogenic bacteria could extend this issue [318–320]. Therefore, probiotic strains must not carry genes that confer resistance to antibiotics, mitigating the risk of propagating antibiotic-resistant strains. Navigating the complexities inherent in ensuring the safety of functional probiotic-based foods and beverages becomes even more challenging amidst an expanding market. As the demand for functional foods surges, including probiotic-rich options, the need for rigorous safety assessments amplifies. The dynamics of a rapidly growing market pose challenges in monitoring and regulating the diverse array of probiotic products flooding the shelves. Harmonizing safety standards across different regions and countries adds another layer of complexity. Varied administrative frameworks and divergent policies regarding functional probiotic-based foods and beverages contribute to inconsistencies in safety assessments and approvals [8]. This patchwork of regulations complicates efforts to ensure uniform safety standards, leaving room for discrepancies in the quality and safety of products available to consumers [20].

Moreover, the inherent nature of live microorganisms in probiotics presents unique challenges in ensuring their stability and viability throughout the food processing and storage phases. Maintaining the viability of probiotic strains until consumption is essential to ensure their efficacy. Factors such as processing conditions, packaging, storage, and shelf life play important roles in preserving the viability of probiotics, adding intricacies to the safety considerations [314]. The expanding market also opens the door to potential misinformation and false claims regarding functional probiotic-based foods and beverages. With the lack of standardized guidelines and stringent regulations, consumers may be susceptible to misleading information about the health benefits of these products. Striking a balance between encouraging innovation and safeguarding consumers from misleading claims becomes a delicate task in this evolving landscape. The principle of “*do more good than harm*” guides the application of probiotics in foods, ensuring their safety amid the emergence of novel strains presents multifaceted challenges. Safety considerations encompassing strain origin, absence of harmful attributes, and the avoidance of antibiotic resistance genes form the cornerstone of safety evaluations. However, complexities arising from market expansion, diverse administrative frameworks, and the inherent nature of live microorganisms demand concerted efforts to establish and enforce robust safety standards in the realm of probiotic-based foods.

#### 5.1.1. Safety Considerations of Probiotic Cohabitation in the Human Gut

The human gut microbiota, composed mostly of nonpathogenic microorganisms, plays a crucial role in maintaining symbiotic host relationships and supporting immunity against pathogenic invasion. The disruption of this balance, known as dysbiosis, has been linked to various human diseases, including anxiety, depression, hypertension, cardiovascular diseases, obesity, diabetes, inflammatory bowel disease, and cancer [321]. The mechanisms through which gut microbiota influence health and disease involve complex interactions between microbial species, metabolic products, and the host immune system [322]. Although these interactions remain incompletely understood, recent clinical studies highlight the significant role of specific microbial species in maintaining gut homeostasis and overall health. The introduction of probiotic microorganisms into the human gut aims to promote beneficial microbial balance and improve health outcomes [323]. However, their successful colonization and integration within the existing gut ecosystem are critical to their effectiveness and safety. Large-scale introduction of foreign probiotic strains may exert selective pressure on the native microbiota, potentially disrupting microbial homeostasis [54, 55]. Some probiotic strains may act as competitors, suppressing beneficial resident bacteria, or as predators, leading to the depletion of certain microbial populations [111]. Additionally, some probiotics may struggle to adapt to the diverse conditions within the human gut, limiting their survival and effectiveness. Therefore, rigorous safety evaluations are necessary to ensure that probiotics do not pose unintended health risks.

Table 4 outlines essential parameters that must be assessed for the safe application of probiotics. These include antibiotic sensitivity, nonhaemolytic properties, cytotoxic effects, and coinoculation (antagonistic) properties. By evaluating these factors, researchers and manufacturers can ensure that probiotics are both safe and effective for human consumption, minimizing potential health risks while maximizing benefits. Addressing these factors in the safety evaluation of probiotic-based foods and beverages will enhance their credibility and ensure their beneficial role in human health. Future research should focus on understanding the long-term effects of probiotic cohabitation and its implications for gut microbiota stability.

### 5.2. Consumer Protection and Market Harmonization

A robust administrative framework stands as the cornerstone of consumer protection and market harmonization in the domain of functional probiotic-based foods and beverages. This framework not only safeguards consumers from misleading claims but also fosters a conducive environment for fair competition and innovation while upholding product quality and safety standards. At its core, a well-structured administrative framework serves as a safeguard against deceptive marketing practices and unsubstantiated health claims. In an era where consumer awareness and demand for functional foods are increasing rapidly, the need to shield consumers from false promises and misleading information is most important [5, 186]. Regulations play an important role in ensuring that claims made by functional probiotic-based food and beverage products are scientifically supported and verifiable. Moreover, a robust administrative framework fosters market harmonization by establishing standardized guidelines and benchmarks for product quality and safety [110]. Consistent regulations across different regions and countries streamline trade, facilitating the smooth flow of functional probiotic-based food and beverage products while minimizing barriers to market entry [324]. This harmonization promotes fair competition among manufacturers and cultivates consumer trust by ensuring a level playing field.

Additionally, stringent regulations set a benchmark for quality control and manufacturing standards. By mandating specific requirements and guidelines, regulations compel manufacturers to adhere to prescribed norms in the production, processing, packaging, and labelling of functional probiotic-based foods and beverages [325]. This ensures that consumers receive products that meet defined safety and quality standards, instilling confidence in the products they purchase. However, despite the undeniable importance of a robust administrative framework, challenges abound in its implementation and enforcement. One of the primary challenges lies in the complexity of establishing universally accepted standards for functional probiotic-based foods and beverages. Diverse microbial strains, varying formulations, and differing product compositions pose hurdles in formulating comprehensive regulations applicable across a wide spectrum of products [313].

Furthermore, the pace of scientific advancements and innovation in the field of probiotics often outpaces administrative developments. Novel strains, emerging research findings, and evolving technologies constantly reshape the landscape of functional probiotic-based foods and beverages [50, 326]. Ensuring that regulations remain updated and relevant amidst these rapid advancements requires continual adaptation and flexibility in administrative frameworks. The divergence in administrative approaches and standards across different jurisdictions adds another layer of complexity. Varying interpretations of scientific evidence, differing risk assessments, and cultural differences influence the formulation and implementation of regulations [19, 41, 103]. Harmonizing these disparate regulations to create a cohesive global framework remains a formidable task, demanding international collaboration and consensus-building among administrative bodies [327].

Moreover, the process of substantiating health claims associated with functional probiotic-based foods and beverages presents its own set of challenges. Proving the efficacy of these products through scientific evidence and clinical trials is often intricate and time-consuming [18, 314]. The stringent requirements for scientific substantiation create barriers for manufacturers seeking approval for health claims, hindering innovation and product development. Additionally, the rapidly evolving nature of the probiotics market, coupled with the absence of standardized definitions and classifications for probiotic strains, complicates administrative oversight. This lack of clear definitions can lead to ambiguities in product categorization and labelling, potentially confusing consumers and impeding effective regulation [328].

A robust administrative framework is important in safeguarding consumers and ensuring market harmonization in the realm of functional probiotic-based foods and beverages; however, its implementation faces multifaceted challenges. Addressing these challenges necessitates a concerted effort by administrative bodies, industry stakeholders, and the scientific community to adapt regulations to the evolving landscape while balancing consumer protection, innovation, and fair-trade practices [329]. Despite the complexities, establishing and enforcing comprehensive and adaptable administrative standards remain imperative to foster a thriving, trustworthy probiotic-based food market.

### 5.3. Responsive Administrative Framework

Probiotics, while potentially serving as biological agents for treating gastrointestinal diseases by altering gut microbiota, face obstacles to their application in this field. These hurdles encompass concerns related to safety, resilience under stress, quantifying postcolonization effects, and the development of evaluation models. Obtaining clinical evidence and approvals for probiotics presents a complex challenge, contributing to the variances in standards and regulations across different regions. The complexities inherent in substantiating health claims and acquiring approvals create a landscape where standards fluctuate, hindering a cohesive global administrative framework for probiotics [315, 327, 330]. One of the primary challenges lies in the nature of probiotics themselves. Probiotics are living microorganisms that interact with the human body's complex ecosystem [331]. Establishing their health benefits and efficacy through clinical trials necessitates meticulous study designs, considering factors like strain specificity, dosage, duration, and the target population as well as application of multiple advanced analytical technologies to further understand and accelerate microbiome science [18, 44, 332, 333]. Designing trials that yield conclusive evidence on probiotics effects on human health requires extensive resources, time, and a robust methodology, which often leads to variations in the quality and quantity of evidence available for evaluation [334].

Moreover, the administrative pathway for probiotics often differs from that of pharmaceuticals. The classification of probiotics as food supplements or functional foods rather than medicinal products complicates the approval process [110]. Administrative agencies may have varying criteria for validating health claims associated with functional probiotic-based foods and beverages, leading to inconsistencies in the evidence required for approvals [333]. The absence of standardized guidelines for clinical trials specific to probiotics further amplifies these challenges [18, 314]. The diversity in administrative requirements across different regions poses another hurdle. Administrative agencies in various countries or regions may demand different levels of evidence for health claims associated with probiotics [17]. This leads to discrepancies in the standards and evidence required for approvals, creating a fragmented landscape where functional probiotic-based foods and beverages face varying levels of scrutiny and approval processes.

Furthermore, the scarcity of universally accepted endpoints for probiotic trials adds to the complexities. Defining clear endpoints and parameters for assessing the efficacy of probiotics in promoting health poses challenges due to the multifaceted nature of their effects [12]. Determining standardized markers or outcomes that universally signify the beneficial impact of probiotics on health remains a considerable obstacle in establishing consistent approval criteria [335]. The variability in study methodologies, endpoints, and trial designs contributes to the discrepancies in evidence available for administrative assessments. This variability leads to differing interpretations of scientific data, creating a scenario where administrative bodies may arrive at disparate conclusions regarding the health benefits of probiotics [235, 336]. Consequently, this contributes to the divergent standards and regulations governing functional probiotic-based foods and beverages across regions.

Addressing these challenges demands a concerted effort from the scientific community, administrative bodies, and industry stakeholders [17]. Establishing standardized guidelines for probiotic clinical trials, endpoints, and evidence requirements would foster a more uniform approach in evaluating probiotic efficacy [15, 314]. Collaborative efforts to harmonize administrative frameworks and set clear criteria for health claims associated with probiotics are crucial in creating a more cohesive global landscape, ensuring consistent standards and consumer protection across regions [330]. Only through such efforts can the challenges in obtaining clinical evidence and approvals for probiotics be mitigated, paving the way for a more transparent and regulated market for these beneficial microorganisms [18].

### 5.4. Setting Standards in the Expanding Probiotics Market

The growing probiotics market stands as a testament to the increasing recognition of their potential health benefits. However, amidst this rapid growth, the need for continuous improvement in standards, policies, and regulations becomes imperative to ensure consumer safety, scientifically proven clinical evidence of health-promoting activity, foster innovation, accurate consumer information, effective marketing strategies, and, above all, a quality product that fulfils consumer expectations and harmonize the market [18, 336]. The pace of scientific advancements and the evolving landscape of probiotics demand a responsive administrative framework that adapts to these changes. As research uncovers new insights into probiotic strains, their functionalities, and potential health benefits, administrative standards must keep pace to evaluate and incorporate this emerging knowledge [313, 336]. Continuous improvement in standards ensures that administrative bodies stay abreast of the latest scientific evidence, enabling them to make informed decisions regarding the safety and efficacy of functional probiotic-based foods and beverages [315].

Moreover, the expansion of the probiotics market necessitates refined policies that address the complexities of this evolving industry. With an influx of novel products and diverse formulations, policies need to be comprehensive, encompassing clear guidelines for product classification, labelling requirements, safety assessments, and health claim substantiation [313]. This clarity ensures transparency and consumer trust while promoting fair competition among manufacturers. Regulations governing functional probiotic-based foods and beverages must also undergo continual refinement to accommodate the evolving nature of these products [17]. Establishing standardized definitions and classifications for probiotic strains, setting clear safety criteria, and harmonizing approval processes across regions are important steps toward creating a cohesive administrative framework [8]. This uniformity ensures consistent product quality, safety standards, and consumer information, regardless of geographical location [18].

Furthermore, continuous improvement in regulations is crucial to address consumer concerns regarding misleading claims and misinformation prevalent in the market. Strict enforcement of regulations that mandate substantiated health claims and accurate product labelling protects consumers from deceptive marketing practices [337]. This instils confidence in consumers, empowering them to make informed choices about functional probiotic-based foods and beverages. Additionally, advancements in technology and manufacturing processes necessitate updated regulations that reflect these innovations. Stricter guidelines for production, processing, packaging, and storage of functional probiotic-based foods and beverages ensure the maintenance of product quality and viability. Continual improvement in regulations helps mitigate risks associated with product contamination or deterioration, safeguarding consumer health [338]. According to Ashwell et al. [338], they have highlighted two challenges: (i) improving the clarity of specific health claims and (ii) grasping the implications of misconceptions surrounding products that carry nutrition or health claims.

The pace at which the probiotics market is expanding demands proactive measures to anticipate and address emerging challenges. Collaborative efforts among administrative bodies, industry stakeholders, and the scientific community are essential in identifying gaps in existing regulations and swiftly adapting to meet the market's evolving needs. This collaboration facilitates knowledge sharing, data exchange, and consensus-building, leading to more effective and relevant regulations. Continuous improvement in standards, policies, and regulations is imperative to keep pace with the rapid growth of the probiotics market [17]. Evolving scientific knowledge, technological advancements, and market dynamics necessitate an administrative framework that is agile, responsive, and adaptable. A forward-looking approach to refining regulations ensures consumer safety, fosters innovation, promotes fair competition, and establishes a transparent and trustworthy probiotics market for the benefit of all stakeholders involved.

## 6. Prospects and Future Perspectives

**Figure 1:**
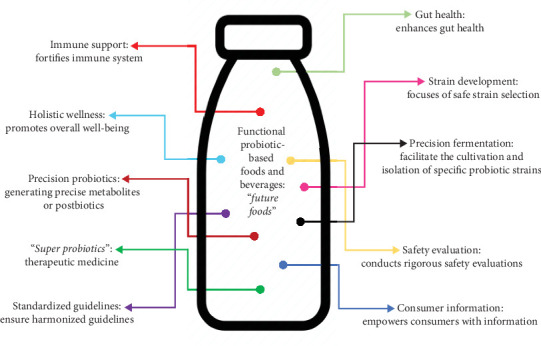
The emergence of functional probiotic-based foods and beverages: exploring the future of food innovation.

The future landscape calls for increased consumer education and awareness. Empowering consumers with accurate information about probiotics' benefits, proper usage, and potential limitations is important in ensuring informed choices and dispelling misconceptions [313]. The trajectory of functional probiotic-based foods and beverages as “*future foods*” is delighted by their potential health benefits and innovation prospects [44]. However, their realization as mainstream dietary choices hinges on ensuring their safety, refining administrative frameworks, fostering scientific advancements, and enhancing consumer education. Embracing these challenges and proactively addressing them will pave the way for a robust, transparent, and thriving probiotics market that aligns with the aspirations of a health-conscious society.  Figure 1 encapsulates the central theme of the figure, highlighting the focus on future-oriented developments in the food and beverage industry with an emphasis on probiotics. The prospects for functional probiotic-based foods and beverages as “*future foods*” are promising, focusing on precision probiotics that target specific health benefits, such as immune support and gut health. Innovations like precision fermentation and the development of “*super probiotics*” are paving the way for more effective, tailored products. Rigorous safety evaluations and standardized guidelines will be essential to ensure consumer trust and the success of these next-generation functional foods in promoting holistic wellness.

### 6.1. Opportunities and Embracing New Technologies

The adoption of new technologies in the realm of functional probiotic-based foods and beverages signifies an era of innovation and advancement within the functional food industry. These emerging technologies offer a spectrum of opportunities, revolutionizing production, preservation, and delivery mechanisms while enhancing the efficacy and appeal of probiotic products [4, 339].

#### 6.1.1. Precision Fermentation

Furthermore, advancements in fermentation techniques, such as precision fermentation, facilitate the cultivation and isolation of specific probiotic strains [340, 341]. Food fermentation stands as one of humanity's earliest methods for food preservation and enhancement, harnessing the power of live microorganisms. Dating back thousands of years, our ancestors unwittingly utilized these microorganisms, only later recognizing their profound health benefits. These beneficial microbes, once discovered, were designated as probiotics. Fermentation is a process rich with untapped secrets, particularly in the realm of unidentified metabolites. Research in this area must continue, especially focusing on traditional, authentic fermented foods and new, innovative fermented products to uncover these hidden potentials. As scientific understanding evolved, researchers delved deeper into the taxonomic and morphological intricacies of these probiotic bacteria. Initially employed solely for preserving surplus food, probiotics have since found diverse applications beyond mere preservation. This precision enables the creation of tailored probiotic formulations, optimizing their functionality and health benefits for targeted applications [342, 343].

#### 6.1.2. Encapsulation Technologies

One notable advancement lies in encapsulation technologies, enabling the protection of probiotic strains through microencapsulation [344, 345]. Innovations like encapsulated and dried probiotics have amplified their benefits, overcoming the challenge of survival in adverse conditions. This technique shields these fragile microorganisms from harsh environmental conditions, ensuring their survival and viability during manufacturing, storage, and even digestion [346–348]. Encapsulation allows for targeted delivery of probiotics to specific areas of the digestive tract, enhancing their efficacy [349, 350]. Moreover, innovative food processing techniques, such as microencapsulation and freeze-drying, have been employed to protect LAB cells from environmental stressors and improve their stability in finished products. Functional probiotic-based foods and beverages enriched with LABs offer a convenient and palatable means to incorporate beneficial bacteria into daily diet. Regular consumption of these products has been associated with a myriad of health benefits, including improved digestive health, enhanced immune function, and potential prevention of gastrointestinal disorders. As consumers become increasingly health-conscious and seek natural alternatives to conventional foods, the market demand for probiotic-rich products is expected to grow exponentially.

#### 6.1.3. Genetic Engineering

Additionally, genetic engineering techniques open doors to engineer probiotic strains with enhanced functionalities or specific health-promoting attributes [351]. The advent of genetically engineered probiotic organisms has ushered in a new era in the nutraceutical industry. These modified probiotics promise heightened benefits for the host, expanding the horizons of health and wellness. These genetically modified probiotics hold promise in addressing targeted health concerns or delivering specialized therapeutic benefits [352–354]. Engineered probiotic products, from fortified yogurts to specialized supplements, showcase these emerging strains' potential to revolutionize the probiotic landscape. In this continuum between tradition and innovation, the historical use of probiotics in foods serves as a testament to humanity's enduring quest for health and longevity. As we embrace both traditional wisdom and cutting-edge advancements, we weave a narrative of probiotics that intertwines the wisdom of the past with the possibilities of the future.

#### 6.1.4. Processing Technologies

Advancements in processing technologies, such as freeze-drying and spray-drying, ensure the preservation of probiotic viability, extending shelf life while maintaining their efficacy in the final products [311, 355, 356]. This preservation allows for the creation of a diverse range of functional probiotic-based foods and beverages, catering to varying consumer preferences. The integration of new technologies into the production and delivery of functional probiotic-based foods and beverages marks an important shift in the functional food industry [8]. These advancements promise enhanced efficacy, targeted delivery, and a broader array of probiotic products, paving the way for a future where these beneficial microorganisms play an increasingly significant role in promoting health and wellness.

#### 6.1.5. Nanotechnology

Nanotechnology emerges as a game-changer, offering novel delivery systems for probiotics [357–359]. Nanosized carriers facilitate the efficient transport of probiotic strains, ensuring their stability and controlled release within the body [360]. These carriers could potentially enhance probiotic absorption, maximizing their beneficial effects. In contemporary medicine, probiotic functional foods have transcended traditional boundaries. They serve as potent nutraceuticals combating multidrug-resistant organisms and even act as efficient transport vectors for therapeutic agents. Looking ahead, the emergence of “*super probiotics*” heralds a groundbreaking chapter in food and therapeutic medicine. These advanced probiotic organisms are poised to redefine human civilization's approach to food and health, promising unprecedented advancements in both preventive and therapeutic care.

### 6.2. Lactic Acid Bacteria as Functional Starter Cultures

LABs have long been recognized for their important role in the microbial fermentation process of various foods and beverages, contributing not only to their flavor and texture but also to their health-promoting properties. These bacteria are instrumental in sustainable food production, addressing environmental, economic, and social concerns [361]. In recent years, there has been a growing interest in utilizing LABs as functional starter cultures in the development of functional probiotic-based foods and beverages. Incorporating LABs as starter cultures in food and beverage production offers a promising avenue to deliver probiotic benefits to consumers while enhancing the sensory attributes of the products. LABs encompass a diverse group of bacteria that convert carbohydrates into lactic acid through fermentation. This metabolic pathway not only preserves the food by creating an acidic environment but also imparts unique flavors and textures to the products. The most commonly employed LAB species in food fermentation include *Lactobacillus*, *Streptococcus*, and *Bifidobacterium*, each contributing distinct characteristics to the final product. Beyond their traditional role in fermentation, certain strains of LABs have been identified for their probiotic properties. Probiotic LAB strains survive the harsh conditions of the gastrointestinal tract and adhere to the intestinal epithelium, where they exert beneficial effects on host health. These effects range from enhancing gut microbiota balance and improving digestive health to modulating immune responses and reducing the risk of certain diseases. Given their resilience and compatibility with the human digestive system, LAB strains are well suited for incorporation into functional probiotic foods and beverages.

The development of probiotic-based foods and beverages has increasingly incorporated mixed-culture inoculums, as multiple probiotic strains often exhibit superior functionality compared to single-strain formulations. Mixed starter cultures enhance microbial diversity, improve gut colonization, and contribute to a more balanced modulation of the host's microbiota. This approach mimics the natural ecosystem of the human gut, where various microorganisms coexist and interact to support digestive and immune functions. Additionally, mixed-culture probiotics demonstrate synergistic effects, improving fermentation efficiency, enhancing sensory properties, and increasing the overall stability of probiotic products. Certain strains may compensate for the weaknesses of others, ensuring better survival in adverse conditions such as acidic environments and bile exposure. However, careful strain selection and compatibility testing are essential to avoid antagonistic interactions. Integrating mixed cultures in probiotic product development not only strengthens health benefits but also enhances the commercial viability of probiotic foods and beverages, making them more effective in promoting gut health. Additionally, by incorporating mixed cultures in probiotic-based foods and beverages production, manufacturers can enhance product stability, improve fermentation efficiency, and provide consumers with more comprehensive health benefits. According to Arslan-Tontul and Erbas [362], the use of mixed cultures in chickpea-enriched boza resulted in the highest cell counts of *L. acidophilus*, *B. bifidum*, and *S. cerevisiae* var. *boulardii* during storage, while gluten addition enhanced protein content fourfold compared to control boza, with fermentation yielding 36 identified compounds, including key fatty acids, and volatile compounds.  Table 5 presents a comparative analysis of the advantages of single-culture and mixed-culture approaches in probiotic production.

The incorporation of LABs as functional starter cultures in the production of functional probiotic-based foods and beverages involves careful selection of strains, optimization of fermentation conditions, and maintenance of viability throughout shelf-life. Advances in microbial biotechnology and food science have facilitated the identification and characterization of probiotic LAB strains with enhanced survival and functionality. Their dual role in fermentation and health promotion makes them invaluable assets in the food industry, paving the way for innovative products that marry taste and wellness. As scientific research continues to unravel the potential benefits of LAB strains and technological advancements enable their successful incorporation into food matrices, the future looks promising for the expansion of functional probiotic-based foods and beverages fortified with these beneficial bacteria. Embracing this synergy between tradition and innovation holds the key to unlocking new opportunities in the ever-evolving landscape of functional foods [6, 186, 311].

The next-generation probiotics are being harnessed for their diverse functionalities, expanding the horizon of functional probiotic-based foods and beverages beyond conventional offerings. A critical aspect contributing to the efficacy of probiotics is their origin, particularly sourcing strains from the human gastrointestinal tract [16, 113]. Probiotics derived from the human gut are inherently well suited to survive and thrive in the intestinal environment [49]. Their compatibility with the human body makes them more effective in exerting their beneficial effects on gut health and overall well-being [182–184]. Yet, as our scientific understanding advances, so does the exploration of novel probiotic strains. Emerging research and technological breakthroughs have led to the discovery and isolation of new bacterial strains, distinct from the traditional fermentative microbes. These emerging strains, carefully selected and cultivated, hold promise in expanding the probiotic repertoire. The integration of these novel strains into modern foods represents an evolutionary leap in the world of probiotics.

### 6.3. Precision Probiotics

It is imperative to acknowledge the dynamic evolution within the probiotics sector, where the subsequent generation of probiotic formulations undergoes meticulous testing, emphasizing a more targeted approach to address specific physiological conditions. Precision functional probiotic-based foods and beverages, a notable advancement in this domain, encompass strains strategically engineered to modulate distinct physiological mechanisms, exemplified by their influence on neurotransmitter synthesis within the cerebral milieu. In the backdrop of contemporary lifestyles contributing to the diminution of microbial diversity within the human gut microbiota, a phenomenon intricately linked to a myriad of health complications, the emergence of precision functional probiotic-based foods and beverages assumes heightened significance in the restoration of this microbial equilibrium. These probiotics exhibit specificity by generating precise metabolites or postbiotics, engaging in targeted interactions with distinct metabolic pathways and immunological processes [102, 236]. The clear potential of precision probiotics lies in their capacity to serve as proactive therapeutic modalities, fostering the preservation of health through the promotion of natural and safe means, thereby attenuating reliance on pharmacological interventions [235]. This strategic utilization may prove important in advancing preventative measures for healthy aging and reducing pharmaceutical dependency.

In the contemporary landscape, a paramount challenge confronting manufacturers lies in elucidating to consumers the nuanced disparities among probiotic formulations, underscoring the crucial discernment that not all probiotics confer equivalent beneficial effects. The imperative to prioritize meticulously researched and scientifically substantiated probiotic strains becomes most important, necessitating a judicious identification and selection process to ensure efficacy and reliability, thereby superseding alternative options. Conventional probiotic administration methods have historically employed pharmaceutical formats, such as capsules and sachets. However, the landscape is swiftly evolving. Presently, viable and robust bacterial strains are incorporated into various comestibles, including prevalent dairy products, juices, ice cream, and chocolate. This transition is facilitated by rigorous and comprehensive testing protocols, given the inherent challenges in preserving the viability and stability of probiotic bacteria within diverse food matrices, particularly when subjected to adverse conditions such as varying temperatures and pH levels.

Alternative modalities garnering considerable attention involve the utilization of nonviable or inactivated bacteria, denoted as postbiotics, owing to their enhanced compatibility with intricate food matrices [103, 174]. Postbiotics represent the resultant products derived from probiotic activities within a fermentation matrix. This encompassing term encapsulates various fermentation components, comprising SCFAs, functional proteins (biopeptides), inanimate microbial cells, cell components, cell-free supernatant, enzymes, exopolysaccharides, microbial cellular functionalities, and other metabolites and substances [102, 103, 174, 236]. These constituents may comprise inactivated probiotics or components thereof, such as bacterial cell walls. Postbiotics manifest naturally in foods subjected to fermentation processes facilitated by live bacterial cultures, exemplified in comestibles like yogurt, cultured milk, kefir, nondairy (plant-based) cultured beverages, tempe (fungal fermented soybean), tapai (fermented white glutinous rice), sourdough bread, kimchi, Japanese natto and miso, soy sauce, pickles, and sauerkraut. The ascendancy of fermented foods in popularity is underscored by their perception as an appealing choice for individuals seeking heightened natural alternatives. These foods confer a diverse array of beneficial bacterial strains upon the consumer, thereby exerting a generally discernible impact on the composition and dynamics of the gut microbiota.

## 7. Essential Factors to Acknowledge

**Figure 2:**
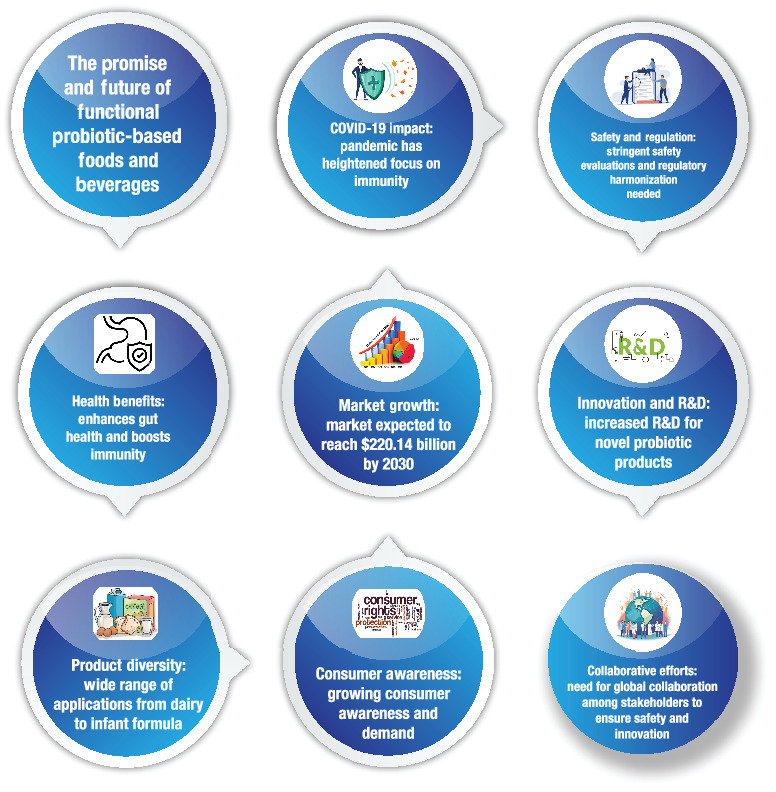
Key drivers and trends shaping the future of functional probiotic-based foods and beverages.

The sustainability efforts within the functional probiotic-based food and beverage sector should prioritize packaging. While the manufacturing process of probiotic ingredients imparts a significant environmental footprint, the primary importance lies in delivering these ingredients through sustainable, plastic-free packaging. Achieving this goal is facilitated by the versatile applications and extensive adaptability of probiotics and postbiotics in present conditions. The principal challenge is bridging the knowledge gap between scientific advancements and consumer awareness. Despite extensive exploration of the gut–brain axis in scientific research, there remains a notable gap in consumer understanding of the inherent connection between the gastrointestinal system and the brain, as well as the profound impact of gut health on cognitive function [115, 226, 293–295, 302, 309]. To enhance the accessibility and efficacy of probiotics for a wider demographic, it is imperative to transcend this cognitive gap by augmenting awareness regarding the mechanistic intricacies of probiotic actions within the human body.  Figure 2 illustrates the key drivers and trends influencing the future of functional probiotic-based foods and beverages. Highlighting health benefits, market growth, consumer awareness, and the impact of COVID-19, it underscores the need for innovation, stringent safety regulations, and global collaboration to meet rising demand and ensure product safety and efficacy.

## 8. Conclusions

In conclusion, the future of functional probiotic-based foods and beverages heralds a transformative era in nutrition, health, and well-being. As these products continue to emerge as potential “*future foods*,” their role in bolstering gut health, fortifying the immune system, and potentially contributing to overall wellness is undeniable. However, the journey toward realizing their full potential is intricately tied to ensuring their safety and navigating the complexities of administrative claims. The safety considerations surrounding probiotics, rooted in sourcing strains from the human gastrointestinal tract and avoiding potential risks, underscore the need for stringent evaluations to safeguard consumers. Concurrently, harmonizing administrative frameworks across diverse regions remains an important challenge, demanding concerted efforts to establish standardized guidelines and transparent practices. Looking ahead, the future prospects of functional probiotic-based foods and beverages hinge on collaborative endeavours to refine safety assessments, adapt to emerging scientific advancements, and streamline administrative frameworks. Achieving this synergy requires a cohesive approach among administrative bodies, industry stakeholders, and the scientific community. As these “*future foods*” continue to evolve, consumer education, innovation in strain development, and global alignment in administrative standards will define their trajectory. Empowering consumers with accurate information, fostering innovation, and ensuring safety through stringent evaluations are important in shaping a future where functional probiotic-based foods and beverages stand as trusted, efficacious, and integral components of a health-conscious society.

## Figures and Tables

**Table 1:** A historical overview of probiotic concept or definitions.

**Year**		**The evolution of probiotics**	**References**
1857	Louis Pasteur (1822–1895)	The pioneer in discovering LABs. He investigated the antagonistic interactions between different bacterial strains, demonstrating the role of nonpathogenic bacteria in controlling pathogens	[29]
1878	Joseph Lister (1827–1912)	Isolated LABs from spoiled milk. He identified a pure culture of *Bacterium lactis*, which is typically found in milk and is responsible for causing lactic acid fermentation	[30]
1890	Ernst Monro	Austrian physician who discovered *Lactobacillus acidophilus*	[31]
1899	Henry Tissier	French pediatrician at the Pasteur Institute in Paris discovered *Bifidobacterium*, a Y-shaped bacterium commonly found in healthy children	[32]
1907	Ellie Metchnikoff (1845–1916)	A bacteriologist at the Pasteur Institute in Paris observed that villagers in the Cucaus mountain of Bulgaria consumed fermented yoghurt drink daily, which seemed to contribute to their long lives. This yogurt was rich in *Lactobacillus bulgaricus*, believed to prevent harmful effects in the large intestine and promote longevity. Ellie Metchnikoff is considered as the father of modern probiotics	[23–25]
1917	Alfred Nissle (1874–1965)	A German physician isolated a new strain of *Escherichia coli* during World War I. He used this probiotic to treat various intestinal diseases, including those caused by *Shigella* and *Salmonella*, and demonstrated its interaction with the body's immune system	[33]
1919	Isaac Carasso (1874–1939)	Recommended yogurt to his patients with gastrointestinal problems. Inspired by this, he started producing yogurt and founded the Danone Company	[26, 22]
1923	Henri Boulard	A French microbiologist discovered that people in Vietnam were using lychee and mangosteen fruit skin for beneficial health properties. He then discovered the probiotic yeast *Saccharomyces cerevisiae* var*. boulardii* in South East Asia to improve gut health and treat diarrhea	[34]
1930	Minru Shirota	Developed the first stable culture of *Lactobacillus casei* strain Shirota and introduced a yogurt-based probiotic drink named Yakult in 1935. This product was launched commercially in Asia and marked a significant advancement in probiotic beverages	[35]
1953	Werner Kollath (1892–1970)	Introduced the term “*probiotika*” to describe active substances that promote health	[36]
1965	Daniel M. Lilly and Rosalie H. Stillwell	Introduced the term “probiotics” as a counterpoint to “antibiotics.” They defined probiotics as substances produced by one microorganism that promote the growth or activity of another microorganism	[37]
1974	R.B. Parker	Refined the definition of probiotics to include “organisms and substances which contribute to intestinal microbial balance.”	[38]
1989	Roy Fuller	Defined probiotics as “live microbial feed supplements that positively influence the host animal by enhancing its intestinal microbial balance,” emphasizing the necessity of live cells for a substance to qualify as a probiotic	[39]
1994	FDA	The Food and Drug Administration (FDA) implemented the Dietary Supplement Health and Educational Act	[40]
2002	FAO/WHO	The Joint Expert Consultation report from the Food and Agriculture Organization/World Health Organization (FAO/WHO) gave the definition of probiotics as live microorganisms which when administered in an adequate amount confer health benefits to the host	[41]
2010	Scientists	Many research studies have focused on the correlation between gut health and gastrointestinal conditions such as irritable bowel syndrome (IBS) and constipation. These studies have also established that not all probiotics offer the same benefits to the host	[42]
2013	Professor Timothy G. Dinan	A specialist in psychiatry investigated the impact of live culture strains on mood and introduced the term “psychobiotics.” His research on the microbiome–gut–brain axis revealed a bidirectional neurohormonal communication system, through which neuroactive compounds play a crucial role in mitigating stress and maintaining homeostasis	[43]
2014	ISAPP	This definition was further endorsed in 2014 by a consensus statement from the International Scientific Association for Probiotics and Prebiotics (ISAPP), confirming its relevance and adaptability for both current and future applications	[44]

**Table 2:** The various types of functional probiotic foods and beverages.

**Probiotic-based foods and beverages**	**Micro-organisms**	**References**
Probiotic fermented milk	*Lactobacillus plantarum* CIDCA 83114, *Streptococcus thermophilus* CIDCA 321, *Kluyveromyces marxianus* CIDCA 8154	[54–56]
Probiotic cheeses	*Lactobacillus salivarius* strains CECT 5713 and PS2	[57, 58]
Probiotic ice creams	*Bifidobacterium animalis* ssp. *Lactis* Bb-12, *Lactobacillus rhamnosus*	[59, 60]
Probiotic butter	*Lactobacillus acidophilus* ATCC 4356, *Bifidobacterium bifidum* ATCC 29521	[61, 62]
Probiotic plant-based beverages	*Bifidobacterium* BB-12, *L. acidophilus* LA-5, *S. thermophilus*	[63, 64]
Probiotic plant-based cheese	*S. thermophilus*, *L*. *fermentum*	[65, 66]
Probiotic fruit juices	*L. plantarum* 299 V, *L. acidophilus* La5	[67, 68]
Probiotic cereal-based beverages	*L. plantarum* (NCIMB 8826), *L. acidophilus* (NCIMB 8821)	[69]
Probiotic fermented vegetables	*L. acidophilus* NCDC 11, *L. plantarum* NCDC 414, *Pediococcus pentosaceus* MTCC 2819	[70, 71]
Functional plant-based fermented beverages	*L. plantarum* 299v	[72, 73]
Probiotic fermented tea beverages (Kombucha)	*L. acidophilus*, *L. casei*, *L. rhamnosus*, *Bifidobacterium lactis*, *Bacillus coagulans*, *K. marxianus*, *S. cerevisiae*, *S. cerevisiae* var. *boulardii*	[74, 75]
Probiotic beer	*Lactobacillus brevis*, *S. cerevisiae* var. *boulardii*	[34, 76–82]
Probiotic mead	*S. cerevisiae* var. *boulardii*	[83, 84]
Probiotic wort-based beverages	*S. cerevisiae* var. *boulardii*	[85, 86]
Yoghurt and fermented milk product	*S. cerevisiae* var. *boulardii*	[87, 88]
Probiotic fermented sausages	*Lactiplantibacillus plantarum* BFL	[89, 90]
Probiotic in bakery	*L. plantarum* P8	[91, 92]
Probiotic and symbiotic chocolate	*L. rhamnosus* GG, *B. breve* DSM 16604, *B. animalis* subsp. *Lactis*, *S. cerevisiae* var. *boulardii*, *B. coagulans GBI-30*, *6086*	[93, 94]
Microencapsulation of probiotics	*L. acidophilus* NRRL B-4495, *Lactiplantibacillus plantarum* NRRL B-4496	[95, 96]
Paraprobiotic preparation for use in foods and beverages	“Inactivated microbial cells (non-viable) that confer a health benefit to the consumer”	[97, 98]
Psychobiotic carried by foods and beverages	“The microorganisms remain viable in concentrations ranging from about 10^6^ to 10^9^ colony form unit (CFU)/mL during processing, storage, and digestion”	[99, 100]
Postbiotic preparation for use in foods and beverages	“Non-viable microbial metabolites or compounds that promote health by exerting biological effects on the host”	[101–103]

**Table 3:** The comprehensive benefits of probiotics for human health and well-being.

**The benefits of probiotics**	**General benefits of probiotics**	**Specific the benefits of probiotics**	**References**
Mental health and well-being	Studies show that probiotics and prebiotics can reduce depression and anxiety symptoms by balancing gut bacteria. They modulate the hypothalamic-pituitary-adrenal axis, optimize gut–brain communication, and generate neuroactive metabolites that influence brain function and behaviour. Additionally, they reduce oxidative stress and inflammatory cytokines, inhibit apoptosis, boost neurotrophic factors like brain-derived neurotrophic factor, protect dopaminergic neurons, suppress neuroinflammation, enhance antioxidant capacity, and produce short-chain fatty acids (SCFAs) linked to improved cognitive function	▪ Improve mental health conditions ▪ Relieving anxiety and depression symptoms ▪ Reducing neurodegenerative disease incidence and enhancing cognitive performance ▪ Improve mood ▪ Emotional well-being and mental state	[118–123]

Eye health	Probiotic eye-drop treatment appears important in exacerbating inflammatory eye diseases	▪ Treat keratoconjunctivitis	[124–126]

Nose health	Probiotics can lessen symptoms and infections and boost sinus health and antibiotic effectiveness	▪ Decreasing nasal cavity infections	[127–129]

Tonsil health	Combining probiotics with antibiotics in the therapy of acute tonsillitis	▪ Preventing tonsilitis	[130–132]

Mouth and teeth health	Utilizing probiotics and postbiotics for oral health benefits. A balanced oral microbiome is essential for preventing the overgrowth of harmful bacteria that can lead to dental issues such as cavities, gum disease, and bad breath (halitosis), which can be due to issues like not cleaning teeth well, gum disease, food stuck in teeth, dirty dentures, broken dental work, mouth cancers, and infections in the mouth, lungs, or stomach	▪ Treatment of oral diseases by balancing mouth microbes and lowering *Streptococcus mutans* in plaque and saliva ▪ Enhancing mucosal membrane function ▪ Minimizing mouth ulcers ▪ Decreasing dental cavities ▪ Combatting gum disease ▪ Minimizing plaque and gingival bleeding (inflammation) ▪ Prevention or cure of periodontal diseases and tooth decay ▪ Treatment for bad breath or halitosis ▪ Prevention of periodontal diseases	[133–146]

Lung health	Research on probiotics indicates enhancements in asthma severity, allergic responses, and immune biomarkers	▪ Fight lung cancer ▪ Prevention of asthma and allergic symptoms ▪ Protection against influenza and reduce length of colds ▪ Diagnosis and treatment of malignant pleural effusion ▪ Managing viral respiratory infections and neuroinflammatory disorders	[147–156]

Preventing respiratory-related diseases	Probiotics help fight respiratory infections by blocking harmful germs	▪ Improve thymus function ▪ Alleviate allergic reactions and respiratory infections	[129, 157, 158]

Liver health	In terms of liver health, probiotics may primarily benefit by inhibiting the production and/or absorption of lipopolysaccharides in the gut, thereby lowering low-grade inflammation levels	▪ Decrease risk of cirrhosis ▪ Reduce endotoxins levels among cirrhosis patients ▪ Decrease kidney stones ▪ Therapeutics strategies in nonalcoholic fatty liver disease ▪ Management of liver fibrosis ▪ Treatment of minimal hepatic encephalopathy and prevents the development of overt hepatic encephalopathy	[159–168]

Increase nutrient absorption	Probiotics can enhance nutrient bioavailability in food and improve gut health for optimal nutrient absorption through various biotechnological techniques. Nutrient absorption mainly occurs in the small intestine, influenced by nutrient bioavailability in food and the human microbiota profile. Modifying the microbiota can be a strategy to alleviate malnutrition	▪ Produce vitamins B2, B6, and B12 and other nutrients ▪ Decrease/cure lactose intolerance ▪ Improve glucose metabolism ▪ Improve protein assimilation and amino acid absorption ▪ Increase calcium absorption and bone health ▪ Increase iron absorption and treatment of anaemia ▪ Enhance the absorption of phytonutrients Improve fibre digestion	[46, 47, 134, 169–175]

Gastrointestinal health	Probiotics, when ingested, are believed to modify imbalanced gut flora or enhance patients' tolerance to their own beneficial bacteria, influencing the development and progression of various gastrointestinal disorders. Several gastrointestinal disorders have been extensively researched concerning the potential benefits of probiotic interventions. These include stomach health, reduce digestive discomfort Reduce digestion discomfort	▪ Prevent the intestine from gastritis and diarrhea ▪ Decreased acute infectious diarrhea and constipation ▪ Decreased infectious diarrhea in both adults and children ▪ Decreased incidence of antibiotic-associated diarrhea ▪ Decreased in risk of *Clostridium difficile* colitis recurrence ▪ An adjunctive treatment for *Helibacter pylori* infection ▪ Reduce abdominal pain and bloating in IBS ▪ Therapy and reduce food poisoning symptoms ▪ Functional gastrointestinal disorders ▪ Treating and managing gastric ulcers ▪ Enhancing and stabilising intestinal barrier efficiency ▪ Improve gastrointestinal dyspeptic symptoms (chronic indigestion) with functional dyspepsia ▪ Effective management of flatulence, treating constipation, acute diverticulitis ▪ Pathogenesis and maintenance inflammatory bowel disease (IBD) ▪ Reduce and inhibit ulcerative colitis ▪ Reduce symptoms of Crohn's disease ▪ Reduce flare-ups of chronic pouchitis	[104, 114, 134, 158, 176–190]

Woman health	Probiotics can defend the vagina against pathogen invasion by blocking attachment sites, generating microbiocidal substances like hydrogen peroxide, maintaining a low pH, and triggering anti-inflammatory cytokine responses in epithelial cells. Additionally, probiotics have shown to be beneficial in addressing dysbiosis related to hormonal changes, potentially impacting the enteric nervous system and gastrointestinal function in early pregnancy	▪ Treatment of urogenital and vaginal infections including yeast vaginitis, bacterial vaginitis, bacterial vaginosis, urinary tract infections, and nonsexually transmitted urogenital infections ▪ Meta-analysis for postmenopausal bone health ▪ Re-establish the pH (3.5–4.5) of the vagina ▪ Prevention of recurrent vulvovaginal candidiasis ▪ Management of cervix cancer ▪ Balance hormones and body mass index in perimenopausal and postmenopausal ▪ Improve quality of life during pregnancy and reduce nausea and vomiting	[134, 191–202]

Supplementation during pregnancy	Current clinical studies explore the potential benefits of probiotic supplementation during pregnancy in protecting against adverse pregnancy outcomes, extending beyond just gestational diabetes mellitus	▪ Hypertensive disorders during gestation ▪ Lipid metabolism dysregulation in pregnancy ▪ Gestational weight gain ▪ Preterm birth ▪ Vaginal microbiota disturbances ▪ Depression and anxiety ▪ Caesarean section ▪ Gastrointestinal dysfunction ▪ Immune system dysregulation ▪ Mastitis	[203]

Skin health	Studies are exploring the connection between gut health and skin issues like aging, wrinkles, and dryness. Probiotics, by balancing the gut and skin interaction, could help manage and improve skin conditions by reducing oxidative stress and inflammation and boosting immune responses	▪ Treat atopic dermatitis in children ▪ Increase burn healing rate ▪ Prevent atopic eczema ▪ Reduce burn infections ▪ Cosmetic and personal care products ▪ Products targeting age-related health concerns	[204–215]

Infant gut microbiome and health	Probiotic supplementation in infancy can positively influence the gut microbiome, aiding digestion and bolstering immune defences. This early microbial balance may reduce the risk of colic, eczema, and other health issues, contributing to overall infant well-being and long-term health outcomes	▪ Treatment of infant's colic ▪ Prevent and/or alleviate childhood rotavirus infections ▪ Establishing a healthy microflora in infants ▪ Therapy to decrease the risk of necrotizing enterocolitis ▪ Improve the gut microbiome in premature infants ▪ Normalize gut colonization for formula-fed infants	[12, 216–224]

Managing gastrointestinal dysfunction in children with autism spectrum disorder (ASD)	Probiotics, like *Lactobacillus* and *Bifidobacterium*, impact the gut–brain axis and may help treat ASD. Many species of probiotics including some single-strain probiotics, probiotic blends of different formulas, and probiotic products, which are often used in combination with dietary interventions or behavioural interventions offering potential health benefits by influencing neuroactive substances like *γ*-aminobutyric acid (GABA) and dopamine. Notably, evidence suggests that probiotics may alleviate gastrointestinal symptoms in children with autism, drawing from both human and animal studies	▪ In clinical studies, probiotics demonstrate potential benefits: (1) improving gastrointestinal dysfunction, (2) correcting dysbiosis, and (3) consequently reducing the severity of ASD symptoms	[225–234]

Immunity and general health	Probiotics play a vital role in supporting immune health by maintaining a balanced gut microbiota. They can enhance the body's natural defences, reduce inflammation, and improve gut barrier function, contributing to overall immune system resilience and better resistance to infections	▪ Strengthening the immune system ▪ Improve sleep quality and length ▪ Generate antibodies specific to the virus ▪ Decrease hypersensitivity ▪ Reduce inflammation and modulate immune system ▪ Improve anxiety or eating disorders (anorexia nervosa, bulimia nervosa, and binge-eating disorders) ▪ Enhanced detoxification rate ▪ Management of osteoarthritis pain ▪ Exhibiting various benefits such as antimicrobial, antibiofilm, antioxidant, anti-inflammatory properties	[10, 11, 134, 235–242]

Human immunodeficiency virus/acquired immunodeficiency syndrome (HIV/AIDS) patients	Interventions in HIV patients aim to restore gut-associated lymphoid tissue (GALT) integrity. Probiotics can enhance gut barrier function, remodel the microbiome, and reduce bacterial translocation and proinflammatory cytokine production, improving immune function even during short-term antiretroviral therapy	▪ Enhance immune response in HIV/AIDS patients ▪ Reduce symptoms in HIV/AIDS patients	[243–246]

Coronavirus disease (COVID-19) treatment	Probiotic strains can reduce virus titres and enhance antibody production, with beneficial impacts noted in COVID-19. Probiotics can also help manage SARS-CoV-2-related complications, including respiratory distress	▪ Improves symptom and viral clearance in COVID-19 outpatients ▪ Immune modulator for the management of COVID-19 ▪ Probiotics strengthening the body's defences against viral threats, including COVID-19 ▪ Probiotics supplements are known to boost immune function and help older people respond better to vaccines ▪ Probiotic bacteria have antioxidant properties that are important for managing COVID-19 because maintaining a balance in redox levels helps control the disease's advancement	[5, 7, 134, 247–250]

Cancer prevention and treatment	Probiotics are increasingly recognized as potential allies in the battle against cancer, demonstrating promising results in both laboratory experiments conducted in vitro and animal studies conducted in vivo. Numerous probiotic strains have shown their capacity to target and combat various types of cancer	Probiotics demonstrated antiproliferative effects against: ▪ Cervical cancer cells (HeLa) ▪ Liver cancer cells (HepG2) ▪ Induce apoptosis in nonsmall cell lung cancer cell ▪ Superficial nonmuscle-invasive bladder cancer ▪ Gastric cancer ▪ Reduce the chance of colon cancer ▪ Oral cancer Preventing and managing mucositis, diarrhea, constipation, nausea, and vomiting in patients undergoing chemotherapy and/or radiotherapy	[134, 251–258]

Metabolic and heart health	The probiotics found in fermented foods contribute to the management of noncommunicable diseases by balancing intestinal microbiota, modulating immune responses, and influencing metabolic processes. Emerging evidence indicates that probiotics, including strains like *Lactobacillus* and *Bifidobacterium*, not only lower LDL-cholesterol, improve the low-density lipoproteins/high-density lipoprotein (LDL/HDL) ratio and reduce blood pressure, inflammatory markers, blood glucose levels, and body mass index but also possess antidiabetic effects by enhancing biomarkers of inflammation and oxidative stress and lowering fasting blood glucose through promoting an anti-inflammatory gut microbiota associated with improved metabolic health	▪ Management of blood glucose ▪ Management of blood pressure ▪ Osteoporosis therapy ▪ Management blood cholesterol ▪ Management of body weight ▪ Therapy management of autism spectrum disorder ▪ Remedy for chronic kidney disease ▪ Cancer alternative prevention and treatment	[130, 227, 231, 259–287]

Preventing food allergies	Probiotics have shown promise in both the treatment and prevention of food allergies. These beneficial microorganisms help modulate the immune system, promoting a balance between proinflammatory and anti-inflammatory responses	▪ Enhancing the gut barrier function, probiotics reduce the likelihood of allergens passing into the bloodstream and triggering allergic reactions	[288, 289]

**Table 4:** Key safety considerations for probiotic cohabitation in the human gut.

**Safety parameter**	**Definition and importance**	**Assessment methods**	**Regulatory guidelines**	**Implications for human health**
Antibiotic sensitivity properties	The probiotic strain should be assessed for its resistance or susceptibility to antibiotics to prevent the spread of antibiotic resistance genes	Antibiotic susceptibility testing (e.g., minimal inhibitory concentration (MIC), disc diffusion)	WHO/FAO guidelines on antimicrobial resistance	Prevents the spread of antibiotic-resistant infections
Nonhaemolytic properties	It is essential to confirm that the strain does not cause haemolysis, which could lead to adverse effects on the host	Blood agar plate assay for haemolysis detection	The European Food Safety Authority (EFSA) safety evaluation criteria	Prevents potential pathogenic effects that may impact overall human health
Cytotoxic properties	Any potential toxic effects on gut epithelial cells or other host tissues should be carefully examined	In vitro cytotoxicity assays on gut epithelial cells	WHO toxicity assessment guidelines	Avoids intestinal damage and inflammation
Coinoculation properties (antagonistic properties)	The probiotic strain's interactions with existing gut microorganisms should be analysed to ensure that it does not excessively inhibit beneficial bacteria	Growth inhibition assays, competition experiments	FAO/WHO guidelines on probiotic interactions	Ensures gut microbiota balance and prevents dysbiosis

**Table 5:** Comparison of single culture vs. mixed cultures in probiotic production.

**Criteria**	**Single culture**	**Mixed cultures**
Microbial diversity	Limited to one strain, reducing functional diversity	Multiple strains provide a broader range of benefits
Gut colonization	May have limited ability to colonize and persist	Higher colonization success due to strain synergy
Health benefits	Provides specific health benefits based on the strain	Offers a wider range of health benefits
Fermentation efficiency	Less efficient due to the single metabolic pathway	More efficient due to complementary metabolic actions
Survivability	More vulnerable to adverse conditions (acid, bile, etc.)	Higher resilience and survival in harsh conditions
Sensory properties	May have a limited impact on flavor and texture	Improves texture, taste, and overall product appeal
Product stability	Less stable due to reliance on a single strain	More stable due to strain interaction and adaptation
Production of bioactive compounds	Limited production of secondary metabolites	Enhanced production of bioactive compounds beneficial for health

## Data Availability

The data is contained within the article.
